# A sustainable electrochemical phosphonylation of phenothiazine. Synthesis of a C-phosphonium betaine with powerful antibacterial activity†

**DOI:** 10.1039/d5ra03690a

**Published:** 2025-07-31

**Authors:** Mahtab Gitipeimay Hamedani, Davood Nematollahi, Ali Goudarztalejerdi, Niloofar Mohamadighader, Farideh Lotfipour

**Affiliations:** a Faculty of Chemistry and Petroleum Sciences, Bu-Ali Sina University Hamedan 65178-38683 Iran nemat@basu.ac.ir; b Planet Chemistry Research Center, Bu-Ali Sina University Hamedan Iran; c Department of Pathobiology, Faculty of Veterinary Medicine, Bu-Ali Sina University Hamedan Iran

## Abstract

To demonstrate the capabilities of electrochemistry in the synthesis of organic compounds, we developed a facile and sustainable direct electrochemical phosphonylation of phenothiazine with triphenylphosphine. In this work, we demonstrated that electrochemically produced phenothiazine-5-ium can react with triphenylphosphine in a water/acetonitrile mixture under simple conditions using graphite and stainless steel electrodes in an undivided cell, galvanostatically and without the use of any catalyst, to synthesize the corresponding C-phosphonium betaine in good yield. The synthesized phosphonium betaine shows strong antibacterial activity against a variety of Gram positive and negative bacteria.

## Abstract 2

A novel C-phosphonium betaine were synthesized *via* electrochemical oxidation of phenothiazine in the presence of triphenylphosphine.

## Graphical abstract

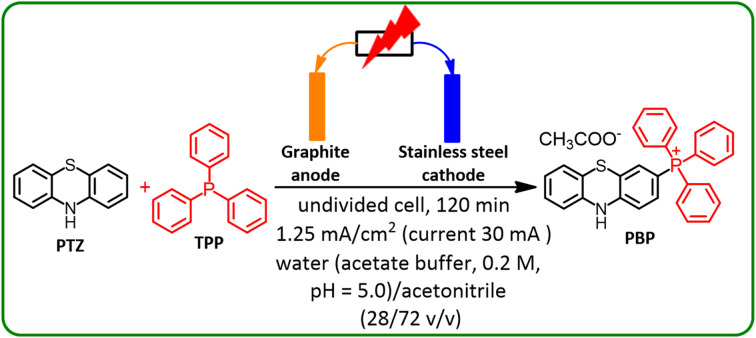

## Introduction

Nowadays, it is essential to develop efficient synthetic methods with high energy efficiency and high atom economy. It is also of great importance to reduce risks by reducing the use of hazardous chemicals and creating inherently safe reactions.^1^ Electroorganic synthesis is compatible with many of the twelve principles of green chemistry. One of the most important features of electrochemical methods is the possibility of replacing hazardous and often expensive redox reagents with electric current in many processes. High selectivity and the fact that electrodes may be considered as heterogeneous catalysts that are easily separated from the products are other unique features of electrochemistry. And finally, it can be concluded that electrosynthesis is a green tool for organic synthesis.^1–10^ Phenothiazine and its derivatives are of considerable interest due to their versatile pharmacological properties. They are used as antipsychotic,^11^ antimicrobial,^12^ anti-malarial,^13^ antiemetics,^14^ anxiety,^15^ antioxidant,^16^ anti-cancer,^17^ antifungal^18^ and antitubercular^19^ drugs.

On the other hand, phosphonium betaines are valuable compounds that exhibit some therapeutic and biological activities.^20–27^ For example, Zhivetyeva *et al.* synthesized some phosphonium betaines from reaction of hexafluoro-1,4-naphthoquinone with triphenylphosphine in dried benzene followed by hydrolysis or treatment with aniline.^20^ They investigated the cytotoxicity of the synthesized phosphonium betaines towards human breast adenocarcinoma, human myeloma, hamster and mouse fibroblasts, as well as their antioxidant and mutagenic effects on a *Salmonella* tester strain and showed that all compounds exhibited comparable IC_50_ values in terms of tumor cell growth suppression.^20^ These properties have led researchers to make great efforts to synthesize phosphonium betaines.^24–31^ In 2015, Mironov *et al.* synthesized some tetraarylphosphonium salts bearing 3,4-dihydroxynaphthyl substituent by refluxing in benzene containing diphenylphosphine and 1,2-naphthoquinone under dry argon atmosphere.^28^ Mahran *et al.* reacted 5-methylisatin with triphenylphosphine in a 1 : 2 molar ratio in dry toluene at reflux temperature and synthesized ylidine triphenylphosphorane.^29^ In 2000, Allen *et al.* synthesized triphenylphosphonium-*p*-toluenesulfonaminido betaine by reaction of triphenylphosphine with 1-bromo-4-*N*-(*p*-toluenesulfonamido)benzene in the presence of nickel(ii) bromide by refluxing in benzonitrile under nitrogen atmosphere.^30^ Furthermore, other examples of the synthesis of phosphonium betaines can be found in ref. 31. These methods have problems such as the use of toxic organic solvents and reagents, harsh reaction conditions, harmful byproducts, and low atom economy.

In order to synthesize new phosphonium betaine derivatives, considering the pharmacological properties of phenothiazines,^11–19^ we decided to synthesize phosphonium betaine based phenothiazine (PBP) and we hope that the synthesized PBP will show new therapeutic properties. In this work, an electrochemical method for the synthesis of PBP is reported. Unlike previously reported methods, this method represents a facile and one-pot electrochemical process for the synthesis of PBP in good yield and purity, galvanostatically in undivided cell in water/acetonitrile mixture under mild and sustainable conditions, without toxic reagents using graphite anode.

## Results and discussion

### Mechanistic studies

**Fig. 1:**
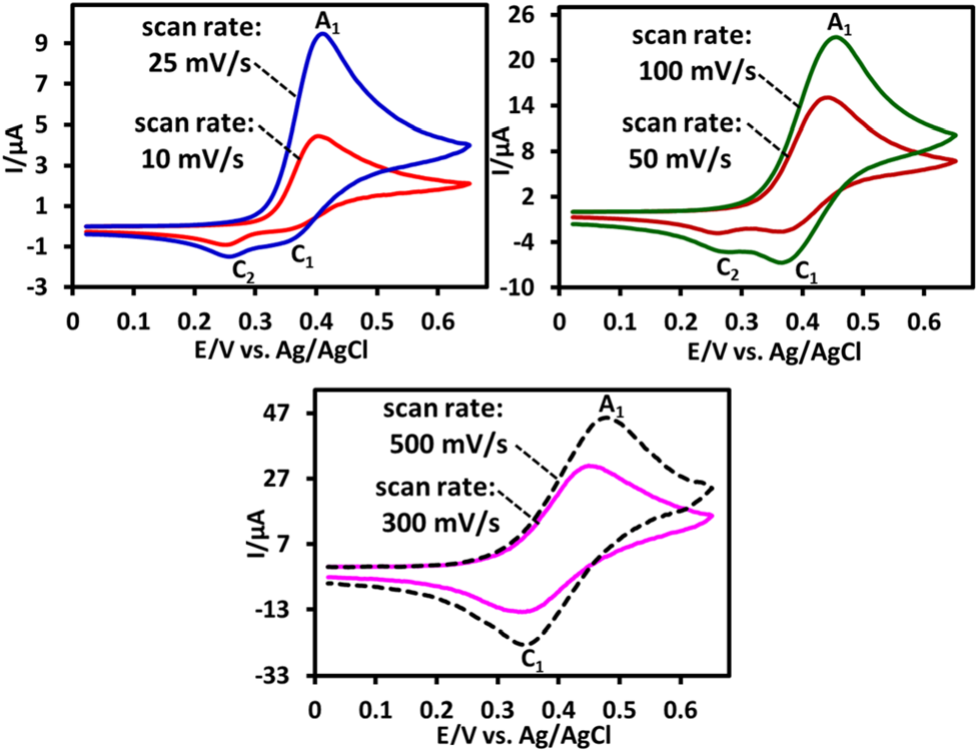
Cyclic voltammograms of PTZ (1.0 mM) in water (acetate buffer, *c* = 0.2 M, pH = 5.0)/acetonitrile (50 : 50, v/v) at glassy carbon electrode at various scan rates. All voltammograms were recorded at room temperature.

**Scheme 1:**
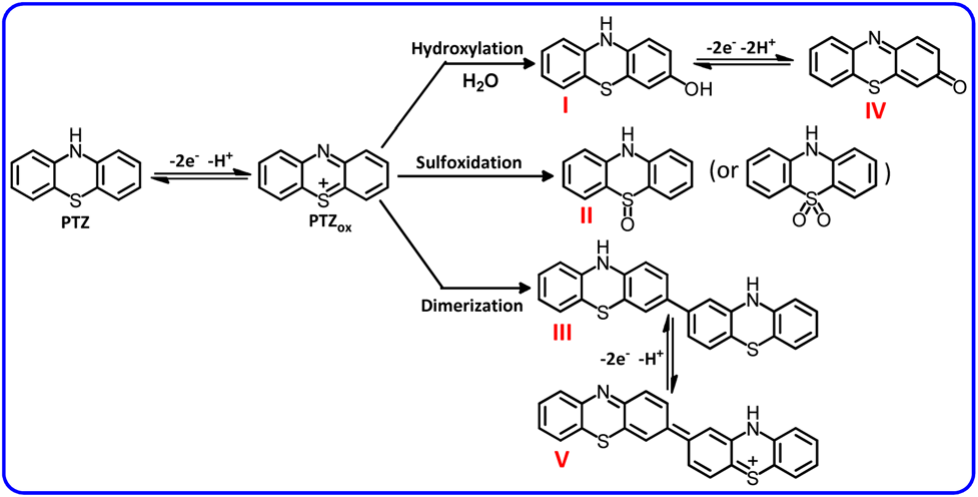
Electrochemical oxidation pathway of PTZ.

The cyclic voltammogram of phenothiazine (PTZ) (1.0 mM) at different potential scan rates is shown in Fig. 1. These voltammograms were recorded in a water (acetate buffer, pH, 5.0, *c* = 0.2 M)/acetonitrile (50 : 50, v/v) mixture and on the glassy carbon electrode surface. In these experiments, acetonitrile was used as a co-solvent to increase the solubility of PTZ. As can be seen, the shape of the voltammogram depends on the potential scan rate. When the scan rate is high (500 mV s^−1^), the cyclic voltammograms show a quasi-reversible process corresponding to the phenothiazine (PTZ)/phenothiazin-5-ium (PTZ_ox_) couple^32^ (Scheme 1).

Conversely, at low scan rates, the cyclic voltammograms exhibit a more complex behavior. In these cases, two cathodic peaks C_1_ and C_2_ are observed in the cyclic voltammograms. Cathodic peak C_1_ is the counterpart of anodic peak A_1_. The ratio of cathodic peak C_1_ to anodic peak A_1_ (*I*^C^_p_^_1_^/*I*^A^_p_^_1_^) depends on the potential scan rate. It increases with increasing scan rate, approaching unity (see 500 mV s^−1^). According to previous studies conducted on electrooxidation of PTZ, PTZ_ox_ is a reactive compound and, depending on the solution conditions, it can participate in the reactions such as dimerization,^33^ hydroxylation,^33^ or sulfoxidation^34,35^ (Scheme 1). However, at high scan rates, PTZ_ox_ does not have enough time to participate in these reactions and therefore PTZ exhibits a quasi-reversible behavior (see 500 mV s^−1^). As can be seen, PTZ_ox_ can be converted to compounds I–III depending on the solution in which the oxidation takes place. It should be noted that the oxidation of compounds I and III is easier than the oxidation of the initial phenothiazine and therefore in the electrolysis cell, they are converted to compounds IV and V, respectively.

**Fig. 2:**
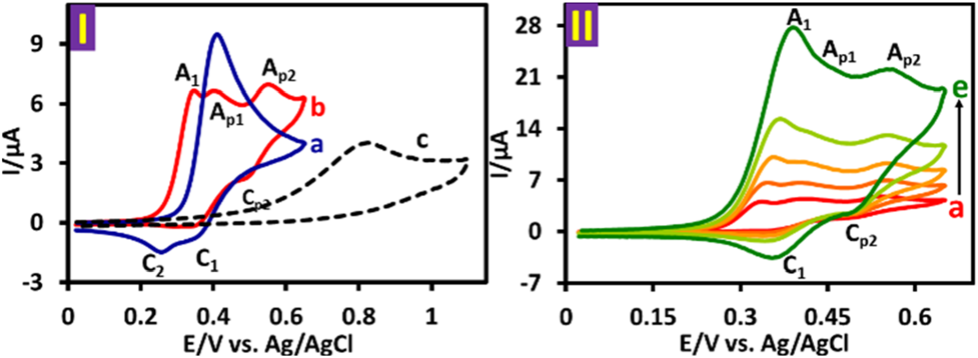
Part I: (a) cyclic voltammogram of PTZ (1.0 mM). (b) Cyclic voltammogram of PTZ (1.0 mM) in the presence of TPP (2.0 mM) and (c) cyclic voltammogram of TPP (1.0 mM). Part II: cyclic voltammograms of PTZ (1.0 mM) in the presence of TPP (2.0 mM) at various scan rates from 25, 50, 100, 250, and 500 mV s^−1^. Solvent: water (acetate buffer, *c* = 0.2 M, pH = 5.0)/acetonitrile (50 : 50, v/v). All voltammograms were recorded at glassy carbon electrode at room temperature.

Fig. 2, part I, curve *b* shows the cyclic voltammogram of a water (acetate buffer, *c* = 0.2 M, pH = 5.0)/acetonitrile (50 : 50, v/v) solution containing PTZ (1.0 mM) and triphenylphosphine (TPP) (2.0 mM). A comparison of this voltammogram with the PTZ voltammogram (curve a) reveals several significant differences resulting from the reaction of PTZ_ox_ with TPP. Under these conditions, three anodic peaks (A_1_, A_p1_ and A_p2_) appear in the anodic sweep, while cathodic peaks C_1_ and C_2_ are completely eliminated and a new cathodic peak (C_p2_) appears at potentials more positive than peaks C_1_ and C_2_. In this figure, voltammogram *c* is for TPP itself, which shows irreversible oxidation peak at a more positive potential than PTZ. Fig. 2, part II, curves *a*–*e* shows that with increasing potential sweep rate, peak C_1_ appears and the current ratio of anodic peaks A_1_ and A_p1_ (*I*^A^_p_^_1_^/*I*^A^_p_^_p1_^) as well as *I*^A^_p_^_1_^/*I*^A^_p_^_p2_^ increases.

**Scheme 2:**
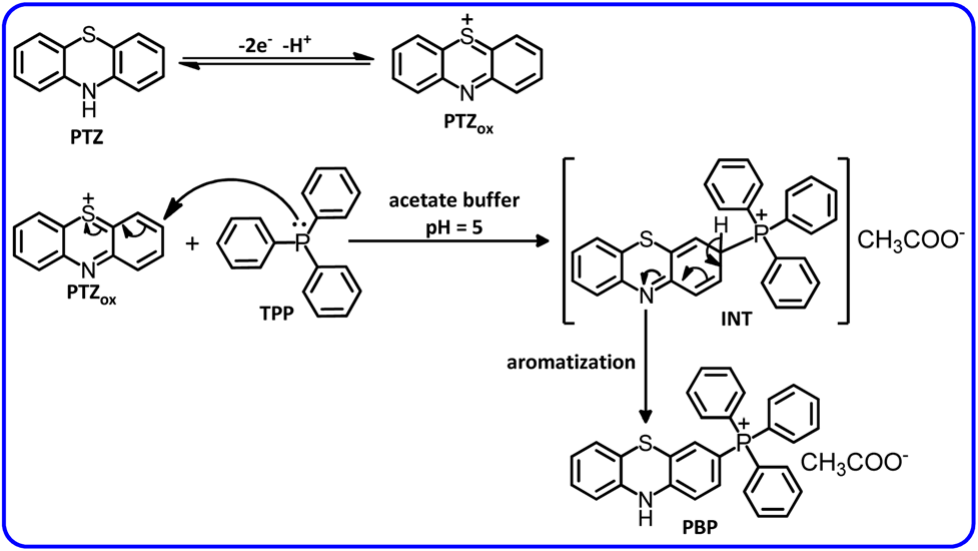
Electrochemical oxidation pathway of PTZ in the presence of TPP.

Considering the obtained electrochemical data as well as spectroscopic data (IR, ^1^H NMR, ^13^C NMR, ^31^P NMR and MS) of the product, we propose the following mechanism for the electrochemical oxidation of PTZ in the presence of TPP (Scheme 2). When a nucleophile such as TPP is present alongside the PTZ_ox_, the reactions introduced in Scheme 1 do not occur and TPP reacts with PTZ_ox_ as an electron acceptor to form the final product. Comparison of cyclic voltammograms *a* and *b* (Fig. 2, part I) shows that in the presence of TPP (voltammogram *b*), the cathodic peaks C_1_ and C_2_ are completely eliminated. This situation indicates that the reaction rate of TPP with PTZ_ox_ is higher than the other reactions introduced in Scheme 1 and the reaction proceeds towards the formation of the desired product (PBP).

**Scheme 3:**
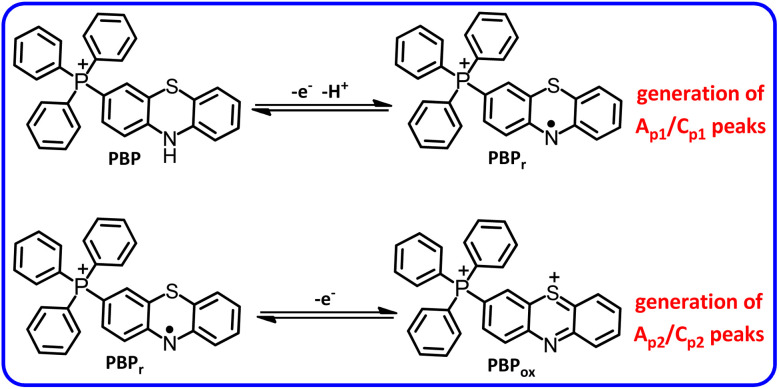
Electrochemical oxidation pathway of PBP.

According to the proposed mechanism, peak A_1_ in curve *b*, is related to the oxidation of PTZ itself, which appears at a less positive potential due to the reaction of its oxidized form (PTZ_ox_) with TPP. Both new peaks A_p1_ and A_p2_ are related to the sequential single-electron oxidation of the product (PBP) (Scheme 3). The presence of the electron-withdrawing group of TPP, as well as the presence of a positive charge, makes the oxidation of PBP more difficult than that of PTZ itself.^36^ The appearance of peak C_1_ at high scan rates is due to the lack of sufficient time for the reaction of TPP with PTZ_ox_. In this condition, the percentage of PTZ_ox_ that did not react with TPP during the cathodic scan is reduced to PTZ.

**Fig. 3:**
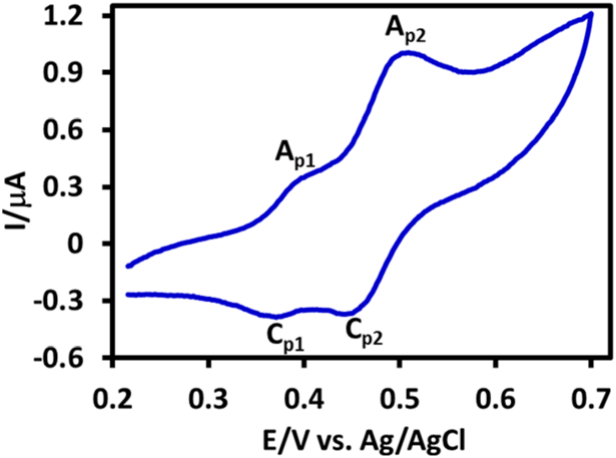
Cyclic voltammogram of saturated solution of PBP in water (acetate buffer, pH 5.0, *c* = 0.2 M)/acetonitrile (50/50 v/v) mixture at glassy carbon electrode. Scan rate: 25 mV s^−1^ at room temperature.

In this work, Fig. 3 shows the cyclic voltammogram of PBP after separation and purification in a water (acetate buffer, *c* = 0.2 M, pH = 5.0)/acetonitrile (50 : 50, v/v) mixture saturated with PBP. In the cyclic voltammogram, two anodic peaks A_p1_ and A_p2_ are observed as well as their cathodic counterparts (C_p1_ and C_p2_). As shown in Scheme 3, these peaks are related to the single-electron oxidation/reduction of redox couples A_p1_/C_p1_ and A_p2_/C_p2_ in reversible and quasi-reversible systems, respectively. The peak potential and peak current ratio of these peaks do not change significantly with changing the potential scan rate. These results complement our previous data on the electrochemical study of PTZ.^33^ In that study, we showed that the oxidation pathway of PTZ differs at different pH values. We reported that while PTZ oxidation proceeds *via* two one-electron steps at pH ≤ 1, this process becomes a two-electron process at 1 < pH ≤ 7 due to the occurrence of the disproportionation reaction. The changes continued with increasing pH, such that the oxidation process at pH values >7 again becomes two one-electron steps. Deprotonation of the radical cation in alkaline solutions and the relative stability of the resulting radical have changed the oxidation of PTZ in alkaline solutions into a two one-electron steps. The same thing happened with PBP, but due to the presence of the electron-withdrawing TPP group and the presence of a positive charge in the PBP structure (increasing acidity), deprotonation of PBP radical cation (PBP˙^+^) occurs at pH 5, and as a result, the oxidation of PBP at pH 5 occurs in two single-electron steps.

### Galvanostatic synthesis

Since constant current electrosynthesis is more comfortable in work, does not require special equipment or expensive supplies, and can be easily performed by non-specialists, this method has been used for the synthesis of PBP, and related parameters affecting the yield and purity of the product have been studied. Here, current density, charge consumption, anode and cathode materials, type and percentage of organic solvent and pH of the solution were optimized using one variable at a time approach. Table 1 shows the variables and their ranges of variation in PBP synthesis.

**Table 1:** Variables affecting the synthesis of PBP

Variable	Value
Current density (mA cm^−2^)	0.41, 0.83, 1.25, 1.66, 2.08
Cathode material	Stainless steel, Al, Cu, graphite, Zn
Anode material	Graphite, Cu, Fe, stainless steel
Water pH	1, 3, 5, 7, 9
Water/acetonitrile %	28/72, 50/50

**Fig. 4:**
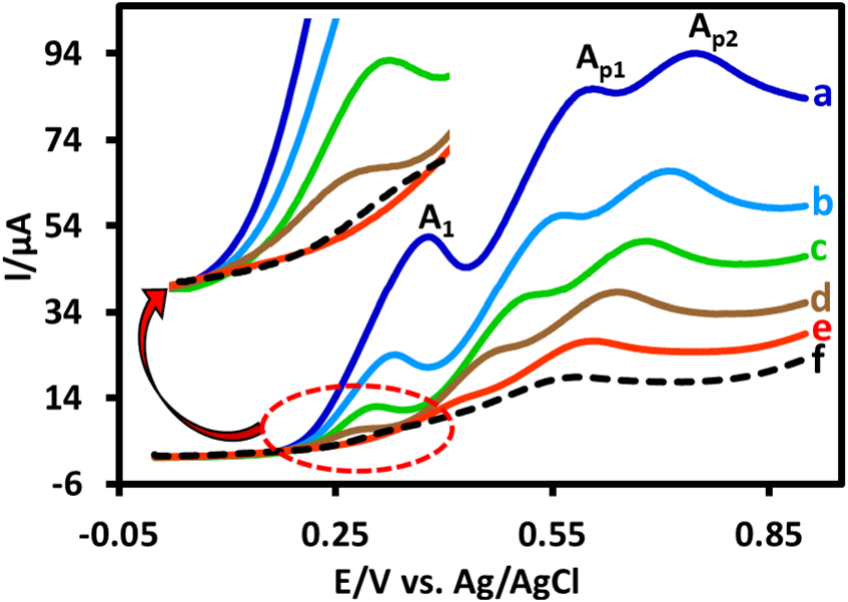
Linear sweep voltammograms of PTZ (1.0 mmol) in the presence of TPP (2.0 mmol) during galvanostatic electrolysis in undivided cell at different times: (a) 0 min, (b) 30 min, (c) 60 min, (d) 90 min, (e) 120 min and (f) 150 min. Applied current: 30 mA (1.25 mA cm^−2^). Solvent: water (acetate buffer, *c* = 0.2 M, pH = 5.0)/acetonitrile (50 : 50, v/v). Scan rate: 100 mV s^−1^. All voltammograms were recorded at glassy carbon electrode at room temperature.

One of the most important parameters that must be optimized first is the amount of electricity consumed. For this purpose, 1 mmol of PTZ was electrolyzed in the presence of 2 mmol of TPP in a water (acetate buffer, pH 5.0, *c* = 0.2 M)/acetonitrile (50/50 v/v) mixture by applying a current of 30 mA in a simple cell equipped with a graphite anode. The linear sweep voltammograms of the solution was recorded after every 30 min (Fig. 4). As can be seen, the A_1_ peak current decreased with the passage of electrolysis time and after 120 min this peak (voltammogram *e*), which is related to the oxidation of PTZ, disappeared. Therefore, the amount of electricity consumed in this synthesis was determined to be 216 coulombs.

This figure also shows that passing more current through the cell reduces the A_p1_ and A_p2_ peaks current (voltammogram *f*), which means that the PBP is oxidized, its impurity increases, and its yield decreases. Considering 216 C electricity as the optimal amount of electricity and since theoretically 193 C electricity (based on Scheme 2) is required to convert 1 mmol of PTZ to PBP, the current efficiency of this synthesis is 89%, which can be interpreted considering the use of an undivided cell and is a good current efficiency for the synthesis of PBP.

**Fig. 5:**
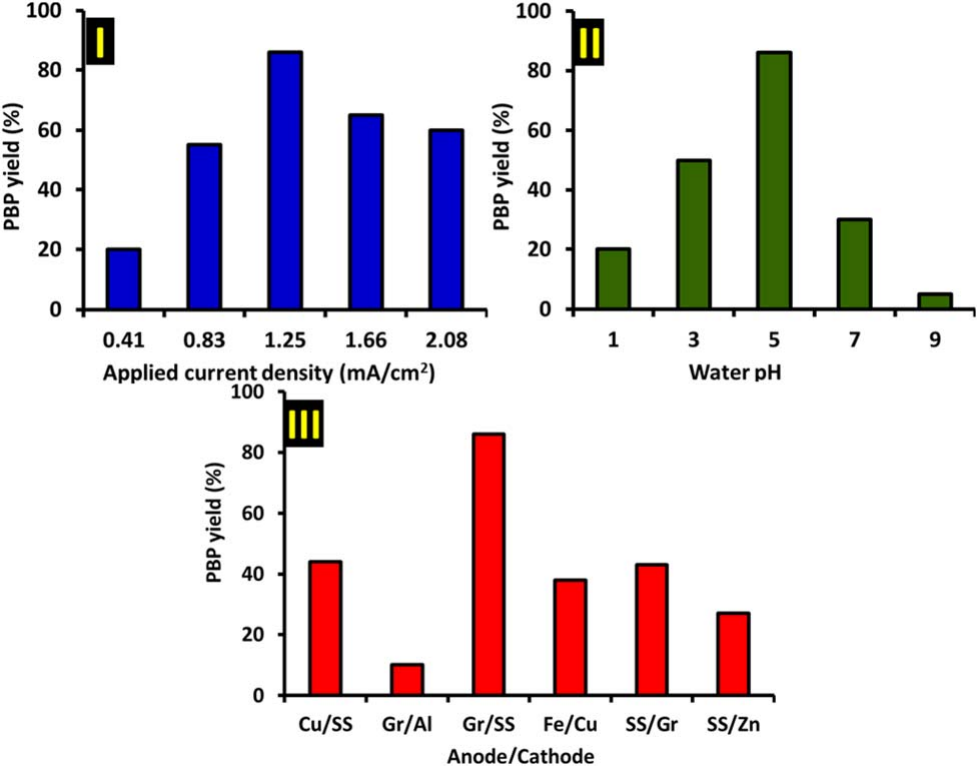
Part I: effect of applied current density on the yield of PBP (electricity consumption: 216 C; anode: graphite; cathode: stainless steel; PTZ: 1 mmol and TPP: 2 mmol) in water (acetate buffer, *c* = 0.2 M, pH = 5.0)/acetonitrile (50 : 50, v/v). Part II: effect of aqueous solution pH on the yield of PBP (applied current density: 1.25 mA cm^2^; other conditions as in part I). Part III: effect of anode and cathode materials on the yield of PBP (conditions as in parts I and II). All experiments were performed at room temperature.

Another important factor affecting the yield of the product, is the applied current density, which is optimized by keeping the electricity consumption constant at 216 C. Fig. 5 part I, shows the variation of PBP yield with respect to the applied current density while keeping other factors constant (PTZ: 1 mmol and TPP: 2 mmol). As can be seen, increasing the current density from 0.41 to 1.25 mA cm^−2^ increases the PBP yield, but further increase in current density causes a decrease in yield. The insufficient overvoltage required for PTZ oxidation at low current densities is one of the factors that reduces the PBP yield. On the other hand, the high and continuous generation of PTZ_ox_ at high current density and its subsequent reaction with TPP decreases the instantaneous concentration of TPP at the electrode surface and increases the possibility of the side reactions introduced in Scheme 1. In addition, reactions such as over-oxidation of PBP, oxidation of TPP and/or solvent are other side reactions that can play a role in decreasing yield when applying high current densities. The results of these experiments are also summarized in Table 2.

**Table 2:** Optimization of effective parameters in PBP synthesis

Entry	Current density (mA cm^−2^)	Cathode material	Anode material	Water/acetonitrile %	Water pH	Isolated yield [%]
1	0.41	Stainless steel	Graphite	28/72	5.0	20
2	0.83	Stainless steel	Graphite	28/72	5.0	55
3	1.25	Stainless steel	Graphite	28/72	5.0	86
4	1.66	Stainless steel	Graphite	28/72	5.0	65
5	2.05	Stainless steel	Graphite	28/72	5.0	60
6	1.25	Stainless steel	Graphite	28/72	1.0	20
7	1.25	Stainless steel	Graphite	28/72	3.0	50
8	1.25	Stainless steel	Graphite	28/72	7.0	30
9	1.25	Stainless steel	Graphite	28/72	9.0	5
10	1.25	Stainless steel	Graphite	50/50	5.0	25
11	1.25	Stainless steel	Cu	50/50	5.0	44
12	1.25	Al	Graphite	50/50	5.0	10
13	1.25	Cu	Fe	50/50	5.0	38
14	1.25	Graphite	Stainless steel	50/50	5.0	43
15	1.25	Zn	Stainless steel	50/50	5.0	27

Another important factor that was examined was the pH of the aqueous solution. In these experiments, aqueous solutions with different pH values and acetonitrile (co-solvent) containing PTZ (1 mmol) and TPP (2 mmol) were electrolyzed at the optimal values of electricity (216 C) and current density (1.25 mA cm^−2^). It should be noted that other factors were kept constant as in previous experiments. The results of these experiments are shown in Fig. 5, part II and Table 2. As can be seen, the PBP yield increases remarkably from 20% to 86% as the pH increases from 1 to 5. However, when the pH value is higher than 5, the PBP yield decreases sharply. Accordingly, pH 5 was chosen as the appropriate pH for the synthesis of PBP. Although TPP is a weak base (p*K*_a_ = 2.73),^37^ its protonation in acidic solutions and subsequent inactivation as a nucleophile is the main factor in the reduction of PBP yield in acidic solutions. On the other hand, competition between hydroxide ion and TPP in basic solutions, over-oxidation of PBP and facilitation of the dimerization process in alkaline solutions, are among the reactions that reduce the yield of PBP in alkaline solutions.

Another factor affecting the yield of the product is the anode and cathode materials. In these experiments, we investigated the efficiency of common and available anodes and cathodes for PBP synthesis (Fig. 5, part III and Table 2). As can be seen, the best results are achieved when graphite and stainless steel are used as the anode as the cathode, respectively.

This method is suitable for gram-scale synthesis. To achieve this goal, it is sufficient to use larger cells and electrodes and perform the synthesis under optimal conditions (Table 2, entry 3). Accordingly, we used an undivided cell (250 mL) equipped with 9 graphite rods as anodes (total area 72 cm^2^) and two stainless steel cathodes with a total area of 14 cm^2^, containing 10 mmol of PTZ (2.0 g) and 20 mmol (5.24 g) of TPP in a mixed solvent consisting of water (acetate buffer, 0.2 M, pH = 5.0)/acetonitrile (28/72 v/v). Electrolysis was carried out at a current density of 1.25 mA cm^−2^ (current 90 mA) for 6 hours, and the product was obtained in a yield of 71%.

### Antibacterial activity

The results of antibacterial activity of the PTZ and PBP are presented in Table 3. As can be seen the phosphonylation of PTZ lead to increase in the antibacterial activity of newly synthesized product (10*H*-phenothiazine-3-yl triphenylphosphonium), PBP, against tested bacteria except *Pseudomonas aeruginosa* and the inhibition zone was increased (Table 3).

**Table 3:** Antibacterial activity of PTZ and PBP

Microorganism	Inhibition zone (mm)	
	PTZ	PBP
*Staphylococcus aureus* ATCC 25923	0	35
*Enterococcus faecalis* ATCC 29212	20	25
*Streptococcus pyogenes* ATCC 19615	20	30
*Escherichia coli* ATCC 25922	20	30
*Salmonella enterica* subsp. *Enterica serovar typhimurium* ATCC 14028	0	25
*Pseudomonas aeruginosa* ATCC 27253	0	0

## Conclusion

We have reported an efficient electrochemical method for the synthesis of a new derivative of C-phosphonium betaine (PBP), which is greener than chemical methods. This method is developed based on the anodic oxidation of PTZ in the presence of TPP in a water/acetonitrile mixture in an undivided cell. The use of an undivided cell and constant current conditions is an important advantage, allowing for easy synthesis on larger scales. This method enables the use of inexpensive and easily accessible graphite and stainless steel electrodes, minimal waste generation, high current efficiency, short reaction times, simple set-up, and avoidance of the use of expensive and toxic oxidizing reagents. Mechanistic analysis supports an electrochemical reaction and a following chemical process in PBP synthesis (EC mechanism). Moreover, PBP was tested for its *in vitro* antibacterial activity against some ATCC bacterial strains and compared with PTZ. It was found that, unlike PTZ, the presence of a TPP group in the PBP structure makes this molecule exhibit good activity against microorganisms. We hope that this electrochemical method will be useful in the synthesis of pharmacophores under mild conditions.

## Experimental section

### Apparatus and reagents

**Fig. 6:**
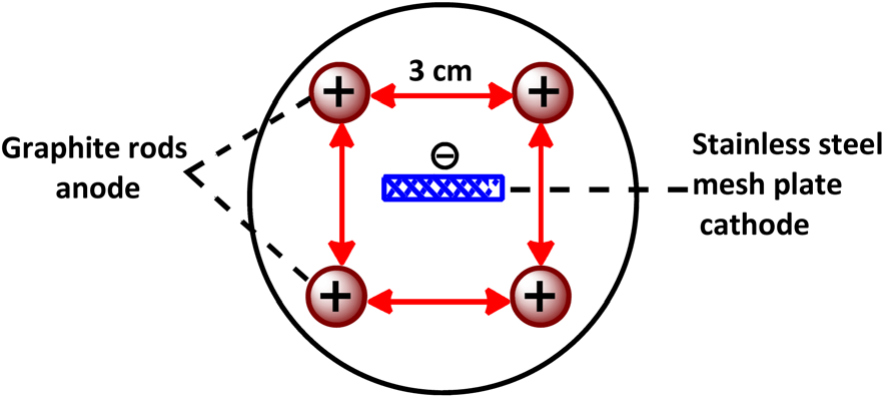
Cell configuration in electrochemical synthesis of PBP.

Cyclic voltammograms were recorded using a potentiostat/galvanostat of the SAMA 500 electroanalyzer system. The working electrode was a glassy carbon disk (diameter 2.8 mm) and a stainless-steel wire was used as the counter electrode. The working electrode potential was measured against Ag/AgCl, 3 M KCl (all electrodes from AZAR electrodes). Phenothiazine and triphenylphosphine as well as phosphoric acid, acetic acid, hydrochloric acid, sodium carbonate, sodium hydrogen carbonate and sodium hydroxide were obtained from Merck and used without further purification. Solvents including chloroform, acetone, acetonitrile, ethyl acetate, *n*-hexane and ethanol were obtained from Merck and Sigma-Aldrich. A pH meter with an accuracy of ± 0.1 pH from Metrohm was used to determine pH. The electrochemical synthesis of PBP was carried out in an undivided cell, equipped with a set of four graphite rods (length 6.5 cm and diameter 10 mm) as anodes and a large stainless steel mesh plate as cathode electrode. The graphite electrodes are placed at the four corners of a square and the stainless-steel electrode is located in the center (Fig. 6). The melting point in open capillary tubes was measured using a model 9100 electrothermal device and there was no change. NMR and FT-IR spectroscopy were performed using a 400 MHz NMR model AVANCE BRUKER DRX 400, a FT-IR model BRUKER-VERTEX 70, and an Agilent mass spectrometer model 5793 Network Mass Selective Detector.

### Electrochemical synthesis of PBP

Electrochemical synthesis of PBP was performed using constant current method. For this purpose, in an undivided cell with a volume of 100 mL, equipped with graphite anode and stainless steel cathode, a mixed solvent consisting of water (acetate buffer, 0.2 M, pH = 5.0)/acetonitrile (28/72 v/v), containing phenothiazine (PTZ) (1.0 mmol) and triphenylphosphine (TPP) (2.0 mmol) is electrolyzed at current density of 1.25 mA cm^−2^ (current 30 mA) for 120 min. At the end of electrolysis, the precipitate was separated from the solution, washed with cold distilled water several times and dried at room temperature under vacuum to give the final product, (10*H*-phenothiazin-3-yl)triphenylphosphonium acetate (PBP), C_30_H_23_NPS^+^CH_3_COO^−^ (light brown solid) in 86% yield. Mp.: 220–221 °C (Dec); IR (KBr) (cm^−1^), 3400, 3245, 2956, 2921, 2851, 1731, 1610, 1580, 1470, 1111, 1074, 1030, 723, 691, 531; ^1^H NMR, (400 MHz, DMSO-*d*_6_) *δ* ppm: 1.73 (s, 3H, methyl), 6.90 (d, 1H, *J* = 16 Hz, aromatic), 7.15–7.35 (m, 2H, aromatic), 7.55–7.65 (m, 2H, aromatic), 7.68–7.85 (m, 14H, aromatic), 7.93–8.00 (m, 3H, aromatic) (see Fig. S1†); ^13^C NMR, (100 MHz, DMSO-*d*_6_) *δ* ppm: 23.1, 115.4, 117.8, 119.0, 123.4, 126.3, 128.1, 128.6, 130.2, 130.4, 131.5, 131.7, 134.3, 134.4, 135.1, 139.5, 148.1, 166.9 (see Fig. S3†). ^31^P NMR (49 MHz, DMSO-*d*_6_) *δ* ppm: 21.3 (see Fig. S4†); MS (*m*/*z*) (EI, 70 EV) (relative intensity): 519 (M + CH_3_COO^−^, <1) 460 (M, 11), 421 (100), 408 (24), 352 (38), 306 (36), 280 (87), 268 (79), 248 (49), 222 (13), 196 (17), 155 (25), 142 (45), 140 (33), 127 (73), 114 (62), 99 (17), 92 (46), 86 (23), 70 (51) (see Fig. S5†). The mass fragments of PBP are given in Table SI.†

### Antibacterial experiments

The antibacterial activity assay of PTZ and PBP was performed using the Kirby–Bauer disc diffusion method according to the guidelines of the Clinical Laboratory Standards Institute (CLSI 2024 (ref. 38)). Briefly, overnight culture of bacterial strains was prepared in Tryptic Soy Broth (Merck, Darmstadt, Germany). The optical density of bacterial suspension at 600 nm (OD600 nm) was near to OD 600 nm of 0.5 McFarland standard. The inoculation (100 μl) of each tested bacterial strain were subcultured on the surface of Mueller-Hinton Agar (Merck, Germany), and Blood agar (for *S*. *pyogenes*) and then the prepared discs were placed on plates. After incubation at 37 °C for 24 h the inhibition zone was measured. Blank disc, Acetonitrile disc, and standard antibiotic disk (ampicillin: 10 μg; tetracycline: 30 μg, and amikacin: 30 μg) were used as controls. All discs were purchased from Padtan Teb^®^ (Padtan Teb Co., Tehran, Iran).

## Ethics approval

This article does not contain any studies with animals performed by any of the authors.

## Consent for publication

We authorize to publish the article without any conflict.

## Author contributions

Mahtab Gitipeimay Hamedani: investigation, formal analysis, writing – original draft. Davood Nematollahi: supervision, project administration, resources, writing – review & editing. Ali Goudarztalejerdi: project administration, investigation. Niloofar Mohamadighader: investigation, writing – original draft. Farideh Lotfipour: investigation.

## Conflicts of interest

The authors declare no conflict of interest.

## Supplementary Material

RA-015-D5RA03690A-s001

## Data Availability

All data generated or analyzed during this study are included in this published article and its ESI† files.
