# Supplementary material for: A sustainable electrochemical phosphonylation of phenothiazine. Synthesis of a C-phosphonium betaine with powerful antibacterial activity
Source: RSC Adv. 2025 Jul 31;15(33):27246–53. doi: 10.1039/d5ra03690a (PMC12311779; doi:10.1039/d5ra03690a)

Supplementary Information (SI) for RSC Advances.
This journal is © The Royal Society of Chemistry 2025

A Sustainable Electrochemical Phosphonylation of Phenothiazine. Synthesis of a C-Phosphonium Betaine with Powerful Antibacterial Activity

Mahtab Gitipeimay Hamedani,[a] Davood Nematollahi,[a,b*] Ali Goudarztalejerdi[c], Niloofar Mohamadighader,[a] Farideh Lotfipour[a]

[a]Faculty of Chemistry and Petroleum Sciences, Bu-Ali Sina University, Hamedan, Iran

[b]Planet Chemistry Research Center, Bu-Ali Sina University, Hamedan, Iran.

[c]Department of Pathobiology, Faculty of Veterinary Medicine, Bu-Ali Sina University, Hamedan, Iran.

[*]Corresponding author. Tel.: + 0098 813 8271541; fax: +0098 813 8272404.
E-mail addresses: *nemat@basu.ac.ir, dnematollahi@yahoo.com* (D. Nematollahi).
Fax: +0098 813 8257407, Tel: +0098 813 8282807.

Figure S1

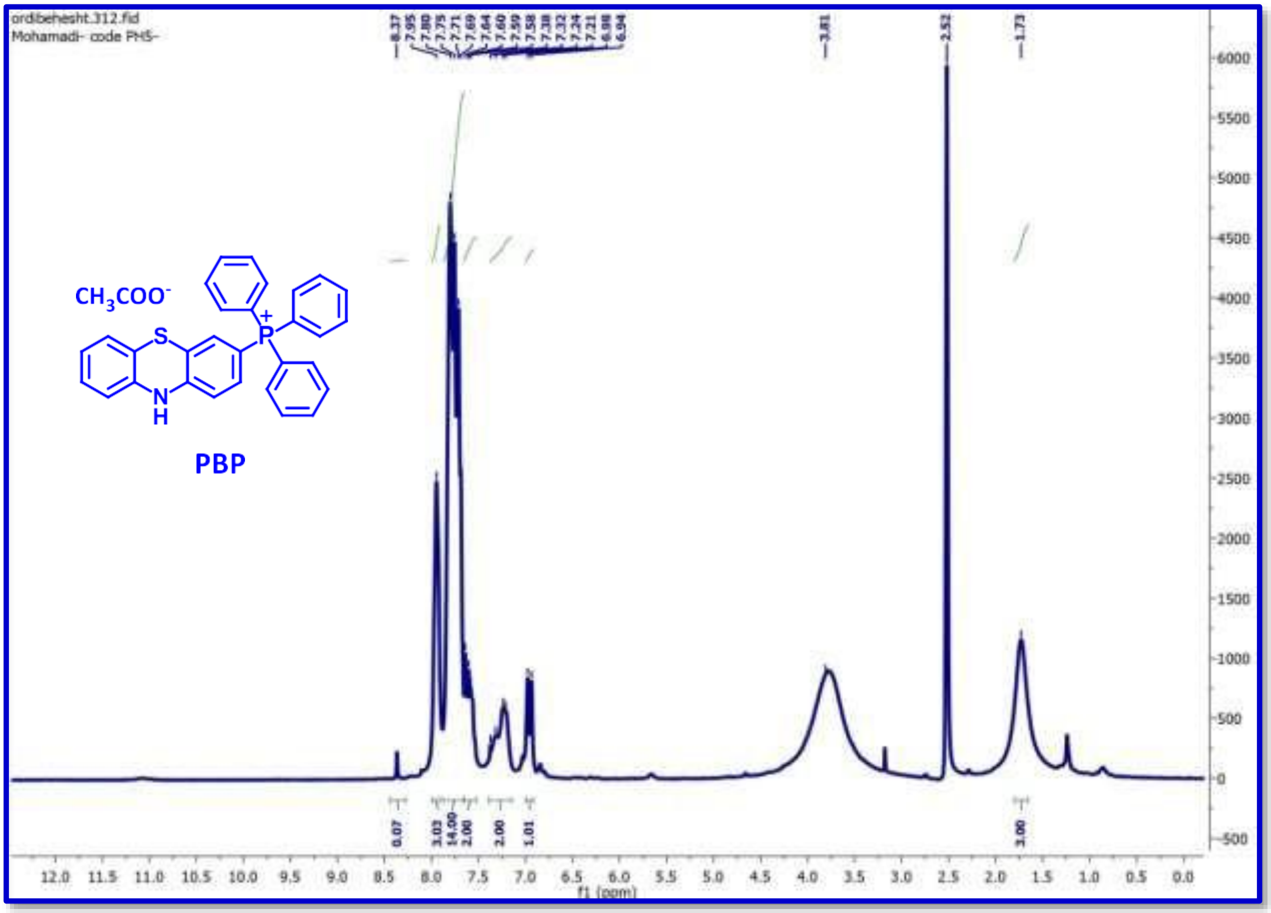
ordibehesht.312.fid
Mohamadi- code PHS-
CH3COO-
S
P
N
H
PBP
f1 (ppm)

Figure S2

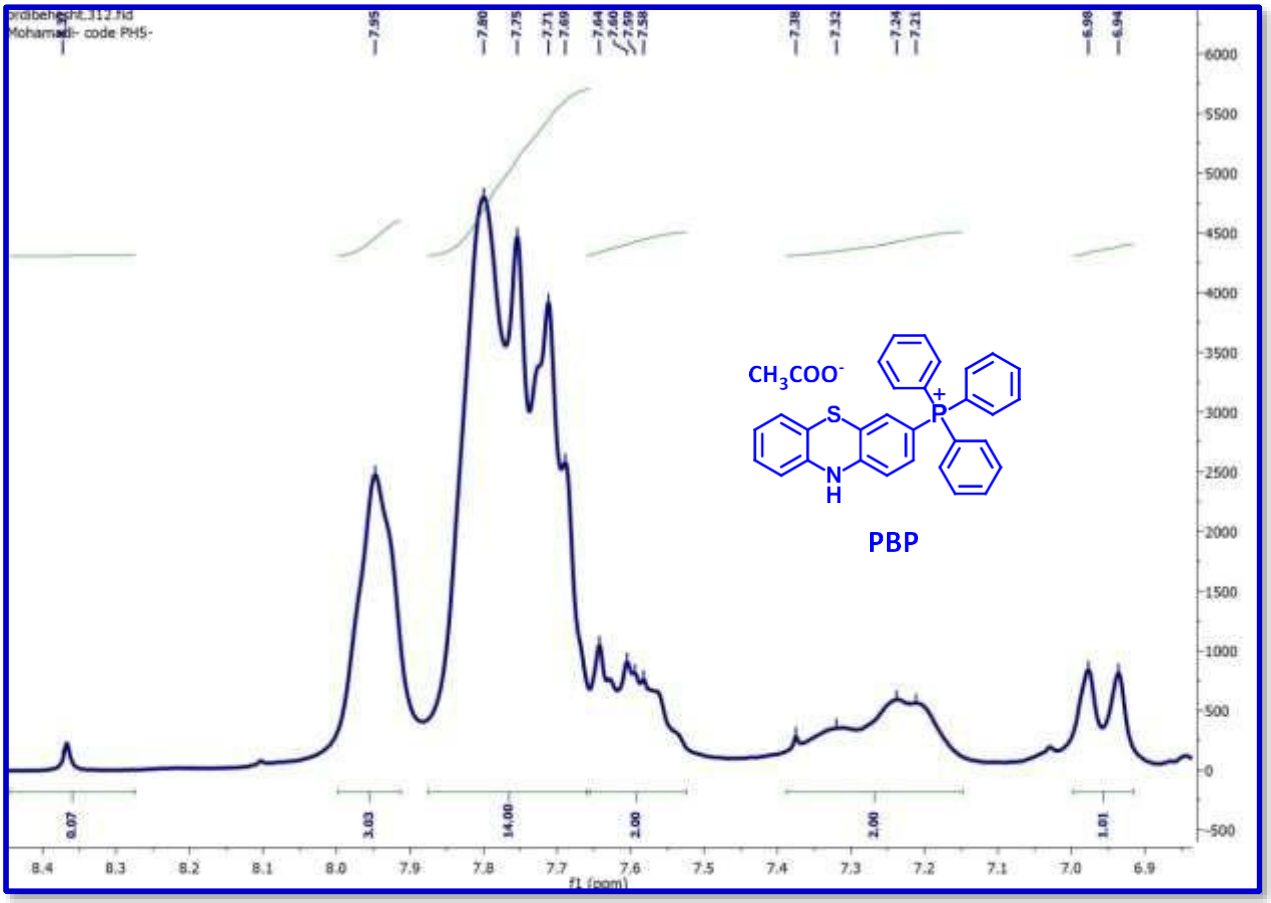

Mohamadi- code PH5-
7.95
7.80
7.75
7.71
7.69
7.64
7.60
7.59
7.58
7.38
7.32
7.24
7.21
6.98
6.94
CH_3COO^-
S
N
H
P
+
PBP
0.07
3.03
14.00
2.00
2.00
1.01
f1 (ppm)

Figure S3

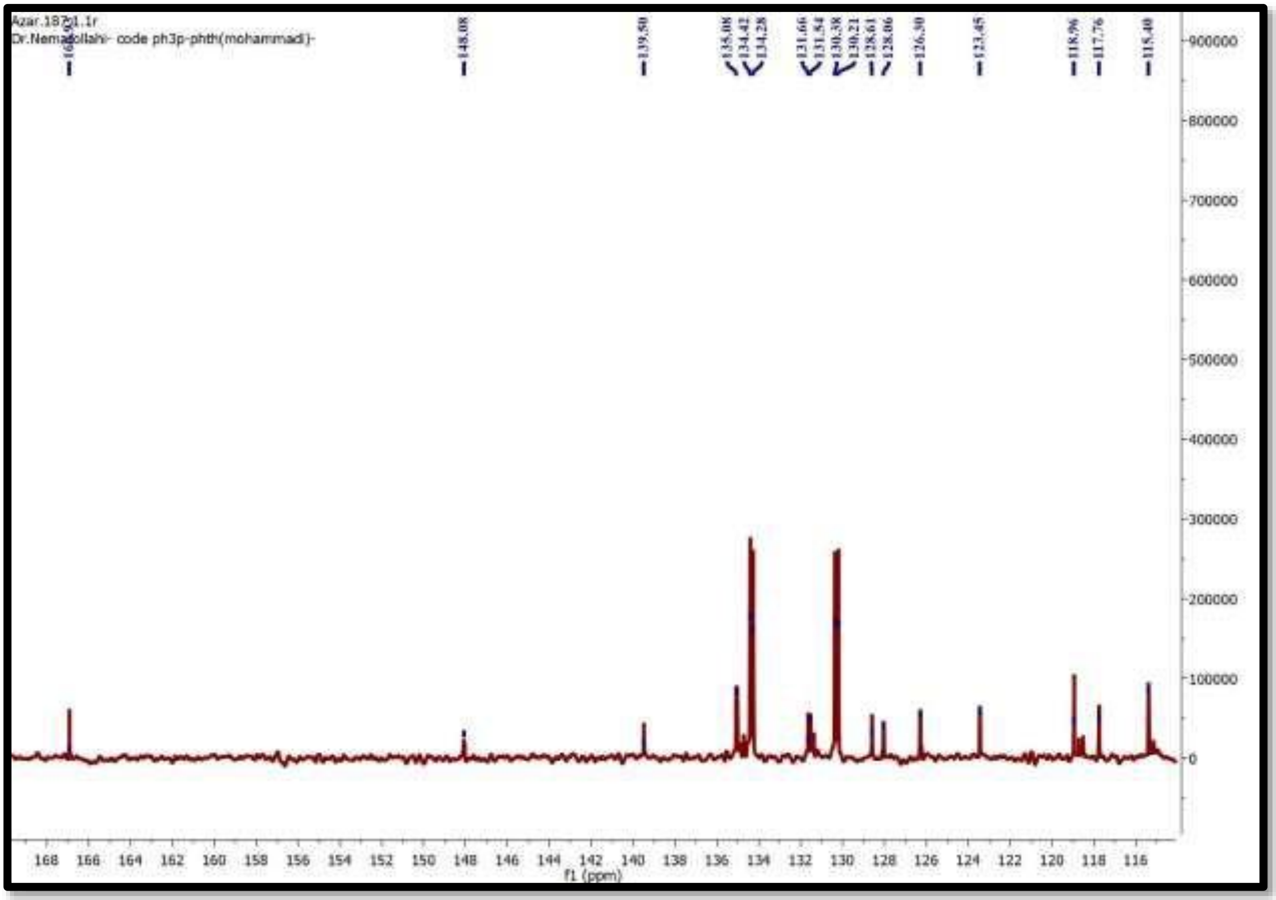
Azar.187.1.1r
Dr.Nematollahi- code ph3p-phth(mohammadi)-
148.08
139.50
135.08
134.42
134.28
131.66
131.54
130.38
130.21
128.61
128.06
126.30
123.45
118.96
117.76
115.40
900000
800000
700000
600000
500000
400000
300000
200000
100000
0
168 166 164 162 160 158 156 154 152 150 148 146 144 142 140 138 136 134 132 130 128 126 124 122 120 118 116
f1 (ppm)

Figure S4

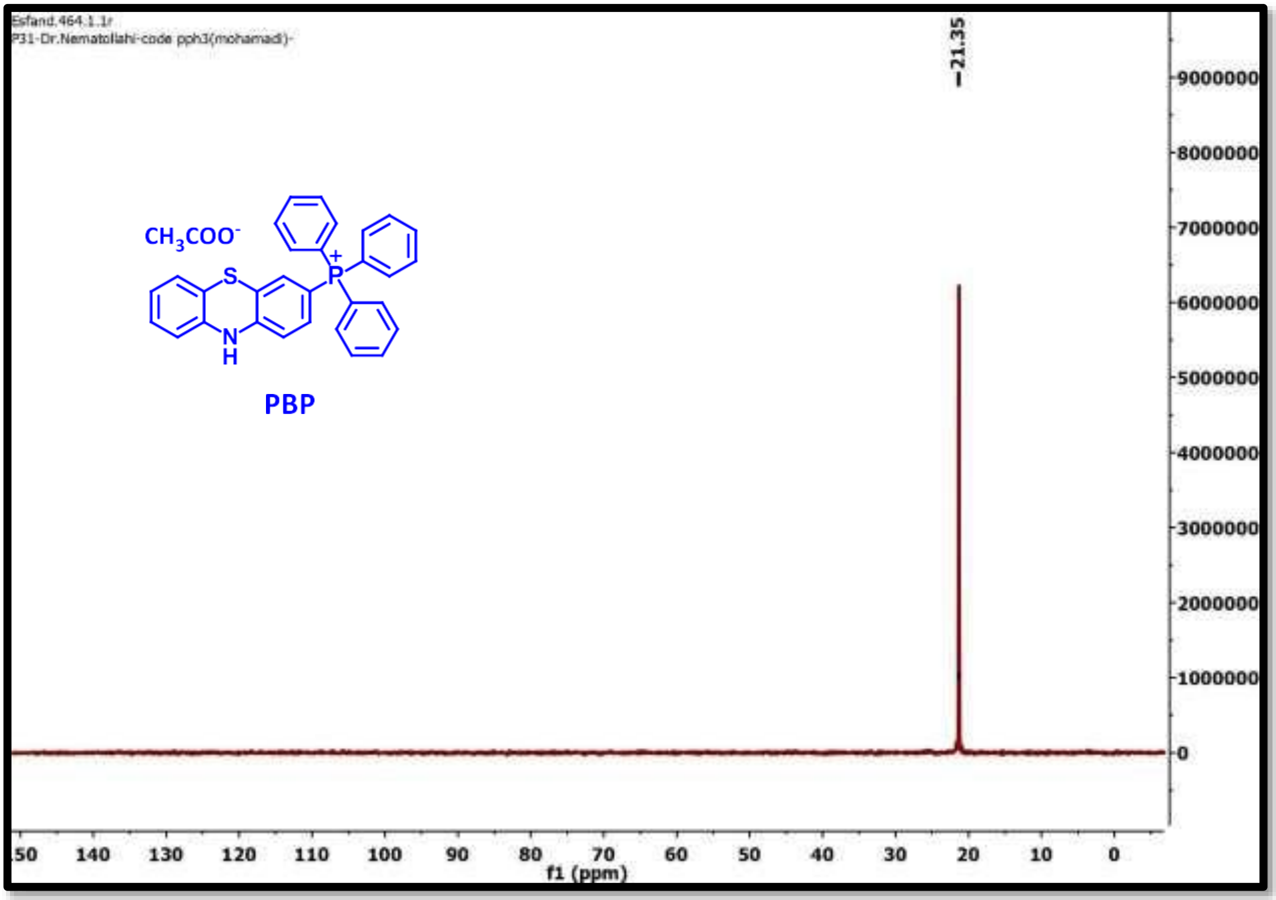
Esfand.464.1.1r
P31-Dr.Nematollahi-code pph3(mohamadi)-
21.35
CH_3COO^-
S
N
H
P
+
PBP
9000000
8000000
7000000
6000000
5000000
4000000
3000000
2000000
1000000
0
150
140
130
120
110
100
90
80
70
60
50
40
30
20
10
0
f1 (ppm)

Figure S5

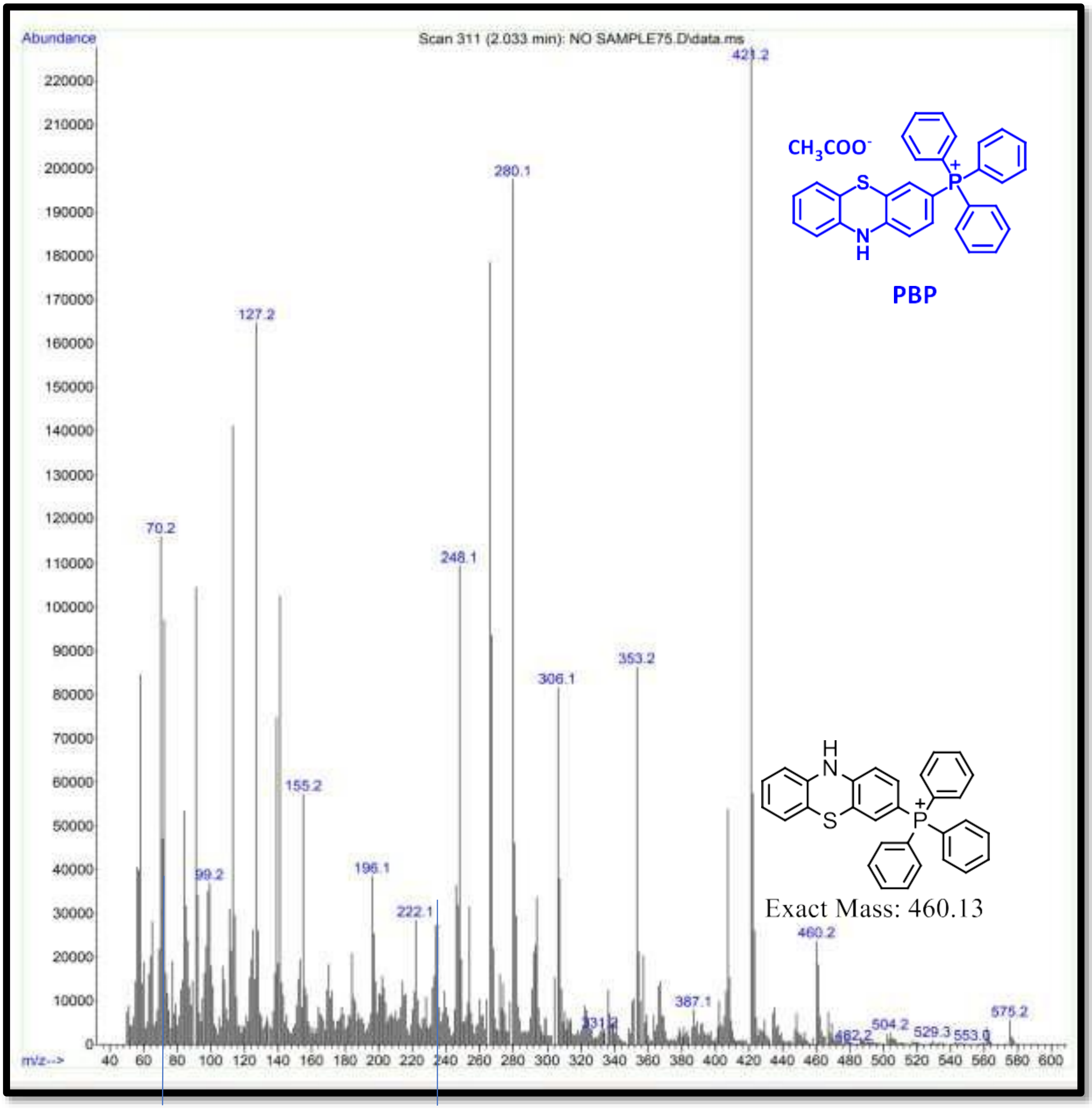
Abundance
Scan 311 (2.033 min): NO SAMPLE75.D\data.ms
421.2
280.1
127.2
70.2
248.1
306.1
353.2
155.2
196.1
99.2
222.1
387.1
460.2
504.2
529.3
553.0
575.2
m/z-->
CH3COO-
PBP
Exact Mass: 460.13

Table S1. Mass fragments of **PBP**.

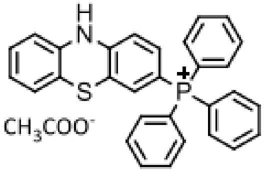 Exact Mass: 519.14	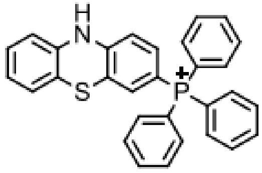 Exact Mass: 460.13	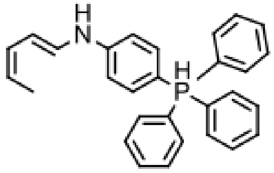 Exact Mass: 421.20
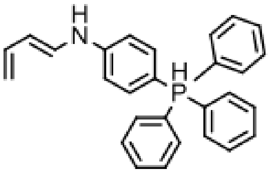 Exact Mass: 407.18	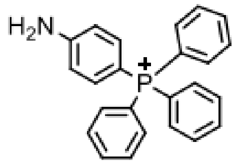 Exact Mass: 354.14	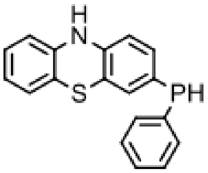 Exact Mass: 307.06
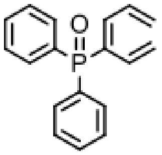 Exact Mass: 280.10	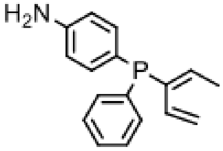 Exact Mass: 267.12	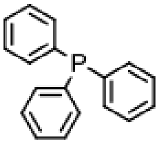 Exact Mass: 262.09
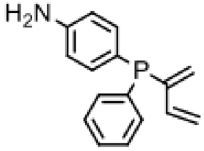 Exact Mass: 253.10	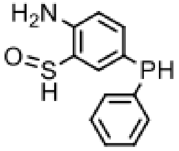 Exact Mass: 249.04	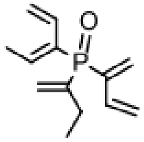 Exact Mass: 222.12
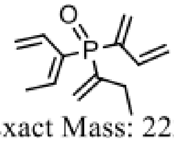 Exact Mass: 222.12	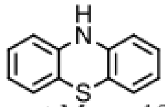 Exact Mass: 199.05	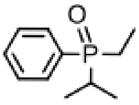 Exact Mass: 196.10
Exact Mass: 182.09 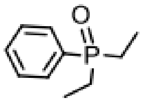	Exact Mass: 154.05 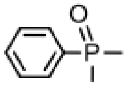	Exact Mass: 140.04 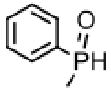
Exact Mass: 128.08 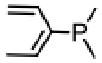	Exact Mass: 127.05 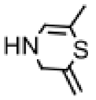	Exact Mass: 124.01 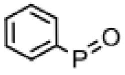
Exact Mass: 114.06 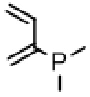	Exact Mass: 113.03 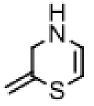	Exact Mass: 113.03 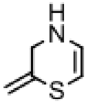
Exact Mass: 100.03 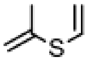	Exact Mass: 90.06 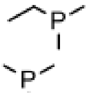	Exact Mass: 90.05 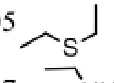
Exact Mass: 76.03 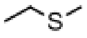	Exact Mass: 76.04 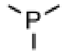	Exact Mass: 71.07 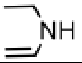

Photo of reaction setup

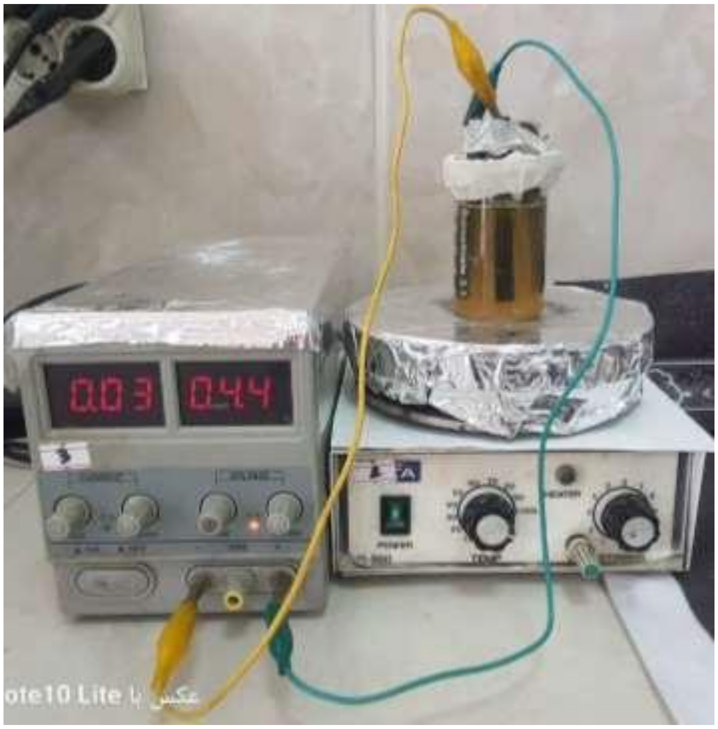

Supplement: RA-015-D5RA03690A-s001 [file RA-015-D5RA03690A-s001.pdf]
